# Associations of fish oil and vitamin B and E supplementation with cardiovascular outcomes and mortality in people receiving haemodialysis: a review

**DOI:** 10.1186/s12882-015-0142-1

**Published:** 2015-08-18

**Authors:** Erica Bessell, Matthew D. Jose, Charlotte McKercher

**Affiliations:** Menzies Institute for Medical Research, University of Tasmania, Hobart, Tasmania 7000 Australia; School of Medicine, University of Tasmania, Hobart, Tasmania 7000 Australia

**Keywords:** Antioxidants, Cardiovascular disease, End-stage kidney disease, Fish oil, Haemodialysis, Omega-3 fatty acids, Vitamins

## Abstract

**Background:**

Cardiovascular complications are the leading cause of mortality in patients with end-stage kidney disease. Research indicates that the Mediterranean diet is protective of cardiovascular disease in the general population. Components of this diet have been trialled in haemodialysis patients with the aim of reducing the risk of cardiovascular disease and improving associated risk factors. Components include fish, fruit and vegetables in the form of fish oil supplements and vitamin and antioxidant supplements. This narrative review provides an overview of observational studies, and interventional and randomised controlled trials examining the association of these supplements with cardiovascular outcomes in haemodialysis patients.

**Methods:**

We reviewed the relevant literature by searching English-language publications in Web of Science and references from relevant articles published since 1992. Eight-seven abstracts were reviewed and 38 relevant articles were included.

**Results:**

The extant literature suggests that risk of mortality is reduced in patients with a higher fish intake and those with higher serum omega-3 fatty acid levels. However, the pathways by which risk of mortality is reduced have not been fully extrapolated. While only a few studies have examined the effect of vitamin B supplementation in haemodialysis patients, these studies suggest that supplementation alone does not reduce the risk of mortality. Finally, studies examining vitamin E supplementation have drawn inconsistent conclusions regarding its pro-oxidant or antioxidant effects. Differences between studies are likely due to methodological variations in regards to dose, route of administration and treatment duration.

**Conclusions:**

Nutritional and dietary supplementation in haemodialysis patients is an area which requires larger, more methodologically robust randomised controlled trials to determine if risk of cardiovascular outcomes can be improved.

## Background

Cardiovascular complications are the most common cause of mortality in people with end-stage kidney disease (ESKD). Dietary approaches in this population have therefore focused predominantly on reducing the risk of incident cardiovascular disease (CVD) and cardiovascular events. Research indicates that healthy adults can reduce their risk of CVD by adopting a diet that is either i) low in fat, cholesterol, saturated fatty acids or sodium or ii) high in fruit and vegetables, fibre, fish, polyunsaturated or monounsaturated fatty acids, or potassium [1]. In healthy populations, dietary interventions focusing on one or more of these strategies have been found to reduce hypertension as well as total and low-density lipoprotein (LDL) cholesterol levels [1]. These dietary interventions may also improve plasma high-density lipoprotein (HDL) cholesterol, triglyceride and antioxidant levels [1].

The Mediterranean style diet incorporates many of these recommendations and is now widely regarded as a beneficial approach to maintaining cardiovascular health throughout life. This dietary approach originated from the traditional diets of Greece, Italy and Spain, and promotes increased intake of high fibre foods such as fruit, vegetables and legumes; increased intake of fish; moderate intake of dairy products and wine (particularly red wine); and low intake of red meats. Previous research generally indicates a positive effect of the Mediterranean diet on traditional cardiovascular risk factors, including blood pressure, and total and LDL cholesterol levels, as well as outcomes including CVD morbidity and mortality [2]. A recent systematic review of 16 relevant studies found that a 2-unit increase in adherence to the Mediterranean diet was associated with a pooled 13 % decrease in risk of CVD (RR 0.87, 95 % CI 0.85–0.90) [3]. Improvements have also been reported in serum markers of inflammation including C-reactive protein (CRP) and interleukin-6 (IL-6) [4].

Given that the Mediterranean diet appears to be cardioprotective in the general population [3], studies have been conducted in people with ESKD to ascertain if any protective effects are offered in this patient population. The most commonly trialled components of this diet in people with ESKD have been fish [5], and fruit and vegetables [6]. However, these two components have mainly been delivered in the form of fish oil supplements, and vitamin and antioxidant supplements. Ensuring adequate intake of vitamins in patients with chronic disease, including ESKD, is important to avoid the subtle effects of low vitamin intake on oxidative stress, and disease occurrence and progression [7]. People receiving dialysis treatment may not meet their daily vitamin requirements for several reasons, including reduced appetite, restrictions on diet (e.g. protein), interference with absorption due to medications, altered metabolic pathways due to uraemia, and losses of water-soluble vitamins during dialysis [7].

The aim of this narrative review is to provide an overview of recently published observational studies and interventional trials examining the association of fish oil, and vitamin and antioxidant supplementation with risk of CVD morbidity and mortality in haemodialysis patients.

## Methods

The relevant literature was reviewed by searching English-language publications in Web of Science and references from relevant articles published from January 1992 to July 2015. Web of Science spans eight science databases, including MEDLINE via PubMed. Literature was searched from 1992 in order to examine contemporary evidence relevant to current treatment practices. Key words (including spelling variants) used in the search included terms relating to: i) haemodialysis (kidney disease, renal disease); ii) study design (intervention, trial, clinical, cohort, observational); iii) CVD morbidity and mortality (cardiovascular, cardiac, mortality, death); iv) omega-3 fatty acids (omega-3, n-3, fish oil, fish, seafood) and v) vitamins and antioxidants (vitamin, antioxidant). Eighty-seven abstracts were reviewed and 38 relevant full-text articles were subsequently included.

## Fish intake and fish oil

Fish, especially oily fish, are a rich source of omega-3 polyunsaturated fatty acids. In humans, the most biologically-important omega-3 fatty acids are eicosapentaenoic acid (EPA) and docosahexaenoic acid (DHA). Omega-3 fatty acids compete with omega-6 fatty acids, such as arachidonic acid and linoleic acid, to be incorporated into the phospholipid bilayer of the cell membrane. Once incorporated into the cell membrane, omega-3 and omega-6 fatty acids can be converted to eicosanoids, such as prostaglandins and leukotrienes [8]. Omega-3 eicosanoids are typically anti-inflammatory, anti-aggregatory and anti-arrhythmic, while omega-6 eicosanoids typically promote inflammation, aggregation and arrhythmia [8]. Omega-3 fatty acids may therefore be protective of CVD, as they also decrease triglyceride levels, blood pressure, heart rate and risk of thrombosis, and  increase HDL cholesterol levels and endothelial function [9]. Examining the role of omega-3 fatty acids in haemodialysis patients is important as many of these patients have hypertriglyceridaemia, which is commonly treated using fish oil supplementation [8].

### Observational studies

Since 2002, six studies have examined the relationship between fish oil and risk of cardiovascular events or mortality in haemodialysis patients (see Table 1). Only two studies have examined the association between dietary intake and risk of mortality. In an early study, incident haemodialysis patients in the United States who reported ‘regular’ consumption of fish in their diet had a 50 % reduced risk of all-cause mortality compared to patients who did not regularly consume fish (adjusted RR 0.50, 95 % CI 0.27–0.90) [10]. However, patients were classified as ‘regular’ fish-eaters if they reported fish consumption during any one of two 3-day food diaries, or during one 24-h food recall, which may not be indicative of the patient’s regular diet. Similarly, another study assessed the effect of omega-6/omega-3 intake ratio on the risk of all-cause mortality in haemodialysis patients using a 3-day food diary [11]. A non-significant trend towards a dose response was observed whereby the risk of 6-year all-cause mortality in patients in the lower quartiles of the dietary omega-6/omega-3 intake ratio was reduced compared to patients in the highest quartile (Quartile 1: HR 0.39, 95 % CI 0.14–1.18; Quartile 2: HR 0.30, 95 % CI 0.09–0.99; Quartile 3: HR 0.67, 95 % CI 0.25–1.79; Quartile 4: Reference; P trend = 0.06) [11].

**Table 1:** Observational studies examining the relationship between fish intake and serum omega-3 fatty acid levels on outcomes in haemodialysis patients

Study	Participants	Follow-Up	Predictor	Outcome	Association
Kutner 2002 [10]. USA	216 incident HD patients (53 % male, aged 20–90 year)	Mean, 3 years	Regular fish eaters versus non-fish eaters	All-cause mortality	RR 0.50 (95 % CI 0.27–0.90)
Friedman 2008 [12]. USA	93 HD patients (66 % male, aged ≥18 years)	Median, 755 days (range 40–770 days)	Omega-3 index < 4.69 % versus omega-3 index > 4.69 %	All-cause mortality	HR 2.48 (95 % CI 0.88–6.95)
Noori 2011 [11]. USA	145 HD patients (57 % male, 53 ± 14 years)	6 years	Quartiles of dietary omega-6 to omega-3 ratio (1) 1.7–7.6, (2) 7.6–9.3, (3) 9.3–11.3, (4) 11.3–17.4]	All-cause mortality	Highest (4): Reference (1.00); (3): HR 0.67 (95 % CI 0.25–1.79); (2): HR 0.30 (95 % CI 0.09–0.99); (1): HR 0.39 (95 % CI 0.14–1.18)
Hamazaki 2011 [13]. Japan	176 HD patients (55 % male, 64 ± 12 years)	5 years	Erythrocyte DHA levels > 8.1 % versus DHA levels < 7.2 %	All-cause mortality	HR 0.43 (95 % CI 0.21–0.88)
Terashima 2014 [14]. Japan	As176 HD patients (55 % male, 64 ± 12 years)	10 years	Erythrocyte DHA levels > 8.1 % versus DHA levels < 7.2 %	All-cause mortality	HR 0.52 (95 % CI 0.30–.0.91)
Friedman 2013 [16]. USA	*N* = 100 (died in 1^st^ year of HD, 58 % male, 67 ± 14 years) *N* = 300 (survived 1st year of HD, 58 % male, 66 ± 14 years)	Retrospective analysis	Quartiles of serum omega-3 fatty acid levels [(1) 1.3 %–3.1 %, (2) 3.1 %–3.8 %, (3) 3.8 %–4.5 %, (4) 4.5 %–15.1 %]	Sudden cardiac death	Lowest (1): Reference (1.00); (2): OR 0.37 (95 % CI 0.17–0.79); (3): OR 0.22 (95 % CI 0.09–0.51); (4): OR 0.20 (95 % CI 0.08–0.51)
Shoji 2013 [15]. Japan	517 HD patients (63 % male, aged 61 [IQR 54–68] yrs)	5 years	Quartiles of serum omega-3 to omega-6 ratio [medians: (1) 0.73, (2) 1.02, (3), 1.27, (4) 1.68]	Cardiovascular events	Highest (4): Reference (1.00); (3): HR 1.35 (95 % CI 0.86–2.04); (2): HR 1.31 (95 % CI 0.85–2.02); (1): HR 1.93 (95 % CI 1.27–2.95)

*Abbreviations*: *DHA* Docosahexaenoic acid, *CI* Confidence interval, *HD* Haemodialysis, *HR* Hazard ratio, *OR* Odds ratio, *RR* Relative risk

A further four studies examined the associations between serum omega-3 fatty acid levels and risk of all-cause or CVD mortality, or cardiovascular events. A cohort study based in the United States found that the risk of mortality after a median follow-up of roughly two years was 2.5 times higher in those with a lower omega-3 index compared to those with a higher omega-3 index (HR 2.48, 95 % CI 0.88–6.95) [12]. While not statistically significant, the cohort included only 93 patients, limiting its statistical power. In a cohort of 176 Japanese patients divided into tertiles according to erythrocyte DHA levels, those with the highest DHA levels had a 57 % lower risk of mortality at 5-years (HR 0.43, 95 % CI 0.21–0.88) [13] and a 48 % lower risk of mortality at 10-years (HR 0.52, 95 % CI 0.30–0.91) [14] compared to those in the lowest tertile. Likewise, in a larger cohort of 517 Japanese patients, those in the lowest quartile of serum omega-3/omega-6 ratio had an increased 5-year risk of cardiovascular events compared to those in the highest quartile (HR 1.93, 95 % CI 1.27–2.95) [15]. Similar associations were found in a cohort of haemodialysis patients in the United States, divided into quartiles according to serum omega-3 fatty acid levels. The risk of sudden cardiac death decreased through the quartiles (Quartile 2: OR 0.37, 95 % CI 0.17–0.79; Quartile 3: OR 0.22, 95 % CI 0.09–0.51; Quartile 4: OR 0.20, 95 % CI 0.08–0.51) compared to the first quartile [16]. These associations indicate a dose-response relationship whereby increased consumption of omega-3 fatty acids is associated with decreased risk of cardiovascular events in haemodialysis patients.

The full benefits of fish oil consumption in haemodialysis patients and the mechanisms by which it may reduce risk of cardiovascular events or mortality are not yet completely understood. Studies examining the association of fish consumption with various physiological markers indicate that patients with a higher consumption of fish have higher HDL cholesterol [17] and albumin levels [10], and are less likely to have type 2 diabetes [10], compared to patients with lower fish consumption. Various studies have also examined the association of serum omega-3 fatty acid levels, or erythrocyte DHA levels specifically, with physiological markers in haemodialysis patients. In a small cohort of haemodialysis patients (*N* = 42) in Eastern Europe erythrocyte DHA levels were negatively associated with plasma levels of IL-6 and tumour necrosis factor-α (TNFα) [18]. However, in a cohort of Japanese patients (*N* = 176) erythrocyte DHA levels correlated positively with serum LDL cholesterol levels [13]. Similar results were found in a larger cohort of Japanese haemodialysis patients (*N* = 517), with serum levels of EPA and DHA correlating positively with non-HDL cholesterol levels [15]. Differing associations between markers with serum omega-3 fatty acid levels may be due to ethnic/racial differences between cohorts and/or methodological differences between studies. Ascertaining the mechanisms of action of omega-3 fatty acids requires further investigation.

### Interventions and randomised controlled trials

Recent intervention and randomised controlled trials examining the effect of omega-3 fatty acid supplementation on the risk of CVD mortality, morbidity and relevant physiological markers in haemodialysis patients are displayed in Table 2. Most trials in this area are limited by methodological weaknesses including small sample sizes and short supplementation durations, ranging from eight weeks [18] to four months [19]. Supplementation with omega-3 fatty acids in only 15 haemodialysis patients in the United States improved the fatty acid profile of erythrocyte membranes by reducing the proportion of saturated fatty acids and increasing the proportion of polyunsaturated fatty acids [20]. The majority of the other trials in this area have focussed on changes to markers of inflammation and the lipid profile with varying results [17–19, 21, 22]. For example, one study reported improvements in inflammatory markers such as IL-6 and TNF-α after supplementation with omega-3 fatty acids [18], while in another two studies inflammatory markers remained unchanged after supplementation [19, 21]. Additionally, supplementation with omega-3 fatty acids has been found to decrease triglyceride levels and increase HDL cholesterol levels [17, 22], while another study found that supplementation did not alter triglyceride or total cholesterol levels [21]. The inconsistencies seen in these trials likely reflect the difficulties in generalising results from small sample sizes, short study durations, and differences in dose of EPA (900 mg to 2010 mg) and DHA (250 mg to 960 mg). A 2008 position statement from the National Heart Foundation of Australia recommended a combined intake of 500 mg/day of EPA and DHA to prevent coronary heart disease, and an intake of at least 1000 mg/day to prevent secondary events in people with prevalent coronary heart disease [9]. Additionally, a review of the literature published since the release of this position statement concluded that fish intake only (ideally 2–3 serves per week), and not supplemented omega-3 fatty acids, is beneficial in the primary and secondary prevention of CVD in the general population [23].

**Table 2:** Studies examining the effects of interventions involving omega-3 polyunsaturated fatty acids on outcomes in haemodialysis patients

Study and location	Participants	Design	Intervention	Outcomes
Svensson 2006 [5]. Denmark	*N* = 206 (65 % male); Intervention: *n* = 103 (66 ± 11 year); Placebo: *n* = 103 (68 ± 12 years)	3-month RCT with 24-month follow-up	1700 mg/day n-3 PUFA (45 % EPA, 37.5 % DHA)	Cardiac event and/or all-cause mortality: HR 1.04 (95 % CI 0.72–1.48). MI: HR 0.30 (95 % CI 0.10–0.92)
Svensson 2008 [17]. Denmark	*N* = 206 (65 % male); Intervention: *n* = 103 (66 ± 11 year); Placebo: *n* = 103 (68 ± 12 years)	3-month RCT with 24-month follow-up	1700 mg/day n-3 PUFA (45 % EPA, 37.5 % DHA)	↓ TG in non-fasting patients only
Perunicic-Pekovic 2007 [18]. Eastern Europe	*N* = 42 (55 % male, 55 ± 8 years)	8-week intervention trial	2400 mg/day n-3 PUFA (60 % EPA, 40 % DHA)	↓ IL-6, TNFα; ↑ albumin, Hb, HDL cholesterol
Saifullah 2007 [20]. USA	*N* = 23 (78 % male, 83 % black); Intervention: *n* = 15 (58 ± 12 years); Placebo: *n* = 8 (57 ± 14 years)	12-week pilot RCT	1300 mg/day n-3 PUFA; (52 % EPA, 26 % DHA)	↓ RBC membrane saturated fat content, plasma n-3/n-6 ratio, CRP; ↑ RBC membrane PUFA content
Vernaglione 2008 [21]. Italy	*N* = 24 (65 % male, 65 ± 18 years)	4-month intervention trial	2000 mg/day n-3 PUFA; (45 % EPA, 12.5 % DHA)	↓ systolic and diastolic blood pressure; no change: all laboratory parameters
Zabel 2010 [38]. Australia	*N* = 28 (50 % male, 61 ± 17 years)	12-week intervention trial	3000 mg/day n-3 PUFA; (67 % EPA, 17 % DHA)	↓ white cell count, peptide YY; ↑ vascular cell adhesion molecules
Gharekhani 2014 [19]. Iran	*N* = 45 (56 % male); Intervention: *n* = 25 (57 ± 13 years); Placebo: *n* = 20 (57 ± 15 years)	4-month RCT	1800 mg/day n-3 PUFA; (60 % EPA, 40 % DHA)	↓ mean serum TIBC; ↑ IL-10/IL-6 ratio; no change: Hb, serum iron, TNFα, CRP
Sorensen 2015 [22]. Denmark	*N* = 161 (65 % male); Intervention: *n* = 81 (66 ± 11 year); Placebo: *n* = 80 (68 ± 11 year)	3-month RCT	1700 mg/day n-e PUFA; (45 % EPA, 37.5 % DHA)	↓ TG; ↑ HDL cholesterol; no change: total cholesterol

*Abbreviations*: *CI* Confidence interval, *CRP* C-reactive protein, *DHA* Docosahexaenoic acid, *EPA* Eicosapentaenoic acid, *Hb* Haemoglobin content, *HDL* High density lipoprotein, *HR* Hazard ratio, *IL-6* Interleukin-6, *IL-10* Interleukin-10, *MI* Myocardial infarction, *PUFA* Polyunsaturated fatty acid, *RBC* Red blood cell, *RCT* Randomised controlled trial, *TG* Triglyceride, *TIBC* Total iron binding capacity, *TNFα* Tumour necrosis factor-α

Only one randomised controlled trial, conducted in Denmark (*N* = 200), examined the effect of omega-3 fatty acid supplementation on risk of mortality in haemodialysis patients over a 2-year follow-up. Three months of supplementation with 1700 mg of omega-3 fatty acids daily reduced the risk of myocardial infarction (MI) by 70 % (HR 0.30, 95 % CI 0.10–0.92), but did not reduce the risk of cardiac events and/or all-cause mortality (HR 1.04, 95 % CI 0.72–1.48) [5]. In this same cohort, 27 patients had atrial fibrillation with these patients recording lower serum DHA levels compared to the 173 patients with sinus rhythm [24]. A large increase in DHA levels after supplementation with omega-3 fatty acids in patients with sinus rhythm was associated with a cardioprotective decrease in average corrected QT interval (time taken for ventricular hyperpolarisation and then repolarisation) [24].

## Vitamins and antioxidants

Pooled results from a recent review of the literature suggest that there may be no effect of vitamin or mineral supplementation, including vitamins A, C and E, and folic acid, on the risk of CVD or mortality in the general population [25]. However, adequate vitamin and antioxidant intake is required to maintain proper physiological functioning and reduce levels of oxidative stress in the body. This is vitally important in haemodialysis patients, as they typically have high levels of oxidative stress and may not meet daily vitamin requirements due to malabsorption or losses during dialysis [7]. A 4-week pilot intervention study found that supplementing 21 haemodialysis patients with fruit juice rich in anthocyanin and polyphenol antioxidants resulted in short term reductions in total DNA damage and markers of protein oxidation; and long term reductions in markers of lipid peroxidation [26]. Conversely, an 8-week randomised controlled trial in the United States providing 20 haemodialysis patients with a daily antioxidant cocktail (800 IU vitamin E, 250 mg vitamin C, 100 mg vitamin B6, 10 mg folic acid, and 250 μg vitamin B12) did not improve any of the measured markers of oxidative stress, inflammation and nutritional status [6]. Other randomised controlled trials have focussed predominantly on only one vitamin or antioxidant, rather than combined therapies, in an attempt to determine which vitamins are most important for haemodialysis patients.

### Vitamin E

Table 3 outlines the results of five randomised controlled trials and one intervention trial of supplementation with vitamin E in haemodialysis patients. All studies provided patients with α-tocopherol, with one study additionally supplementing patients with other tocopherols and DHA [27]. This study reported improvements in IL-6 levels and white cell count, however it is unknown to which supplement these improvements can be attributed. Two additional trials reported increases in α-tocopherol and decreases in γ-tocopherol after supplementation with α-tocopherol, indicating that perhaps these two forms of vitamin E are maintained in equilibrium within the body [28, 29]. Neither study observed improvements in markers of inflammation or oxidative stress. A 12-month randomised controlled trial of α-tocopherol supplementation reported a decrease in total antioxidant status and erythrocyte superoxide dismutase activity (an antioxidant enzyme), indicating the complexity of the antioxidant system, as forms of vitamin E can be both pro-oxidant or antioxidant [30]. These different actions of vitamin E may be mediated by route, dose or duration of administration.

**Table 3:** Trials examining the effects of vitamin E supplementation on outcomes in haemodialysis patients

Study	Participants	Design	Intervention	Outcomes
Boaz 2000 [31]. Israel	*N* = 196 with CVD (69 % male); Intervention: *n* = 97 (65 ± 8 years); Placebo: *n* = 99 (64 ± 9 years)	RCT, median length 519 days, range 10–763 days	800 IU/day α-tocopherol	Cardiac events: RR 0.54 (95 % CI 0.33–0.89); MI: RR 0.45 (95 % CI 0.20–0.99); no effect on risk of cardiovascular or all-cause mortality
Smith 2003 [29]. USA	*N* = 11 (55 % male, mean 64 years, range 42–81 year)	2-month intervention trial	400 IU/day α-tocopherol	↑ α-tocopherol, metabolites of α- and γ-tocopherol, post-dialysis haematocrit; ↓ γ-tocopherol; no change: markers of inflammation
Himmelfarb 2007 [27]. USA	*N* = 63 (63 % male); Intervention: *n* = 31 (58 ± 2 years); Placebo: *n* = 32 (61 ± 2 years)	8-week RCT	Daily: 800 mg DHA, and tocopherols (γ 308 mg, α 13 mg, β 11 mg, δ 11 mg)	↑ erythrocyte DHA; ↓ IL-6, WCC, neutrophils; No change: CRP, F2-isoprostanes, protein carbonyls
Lu 2007 [28]. USA	*N* = 27 (44 % male); Intervention: *n* = 13 (31–72 years); Placebo: *n* = 20 (31–86 years)	6-month RCT	800 IU/day α-tocopherol	↑ α-tocopherol; ↓ γ-tocopherol; no change: markers of glycoxidation, lipid peroxidation
Antoniadi 2008 [30]. Greece	*N* = 47 (49 % male); Intervention: *n* = 27 (59 ± 14 years); Placebo: *n* = 20 (60 ± 12 years)	12-month RCT	500 mg/day α-tocopherol	↓ total antioxidant status, erythrocyte SOD activity (antioxidant enzyme)

*Abbreviations*: *CI* Confidence interval, *CRP* C-reactive protein, *CVD* cardiovascular disease, *DHA* Docosahexaenoic acid, *IL-6* Interleukin-6, *IU* International units, *MI* Myocardial infarction, *RCT* Randomised controlled trial, *RR* Relative risk, *SOD* Superoxide dismutase, *WCC* White cell count

Only one randomised controlled trial examined outcomes for patients supplemented with α-tocopherol [31]. In this trial, 196 haemodialysis patients with pre-existing CVD in Israel were provided with 800 IU α-tocopherol or placebo daily until an outcome occurred or the study ended. Patients receiving a supplement had a 46 % reduced risk of total cardiovascular events, including acute MI, ischaemic stroke, incident peripheral vascular disease, and unstable angina (RR 0.54, 95 % CI 0.33–0.89), and a 55 % reduced risk of MI (RR 0.45, 95 % CI 0.20–0.99). However, supplementation with α-tocopherol did not protect against cardiovascular or all-cause mortality [31]. A larger prospective study involving 1046 diabetic haemodialysis patients in Germany, with a median follow-up of four years, found that baseline α-tocopherol levels were positively associated with body mass index, HbA1c (marker of long term blood glucose levels), total cholesterol, LDL cholesterol and triglyceride levels, and negatively associated with male gender, duration of diabetes, history of arrhythmias, and HDL cholesterol levels [32]. For each increase in standard deviation, α-tocopherol levels were associated with a 28 % reduction in risk of stroke (HR 0.72, 95 % CI 0.56–0.94) after adjustment for age, sex, body mass index, albumin, phosphate, HbA1c, and LDL cholesterol. Additional adjustments for triglyceride levels, however, removed this significant association (HR 0.87, 95 % CI 0.63–1.21). The authors concluded that this may have been due to the overall poor nutritional status in patients, resulting in low levels of lipids and vitamins [32].

### Vitamin B

Table 4 describes two randomised controlled trials of vitamin B supplementation in people with advanced chronic kidney disease (eGFR ≤ 30 mL/min/1.73 m^2^) or ESKD. These studies involved large sample sizes, long durations of supplementation [33] or extended follow-up following supplementation [34]. Neither of these studies identified any protective effects of vitamin B supplementation against risk of cardiovascular events or all-cause mortality. One study population, however, was 98 % male and included a majority of patients with advanced chronic kidney disease, prior to development of ESKD [34]. Cardiovascular outcomes and their determinants may be different in individuals with advanced chronic kidney disease, where some kidney function still remains, compared to those with ESKD, where the kidneys are no longer functional. Similarly, a retrospective analysis of 1790 patients enrolled in the Hemodialysis (HEMO) Study in the United States found that folate (vitamin B9) intake did not reduce risk of mortality from stroke at three year follow-up [35].

**Table 4:** Randomised controlled trials examining the effects of vitamin B supplementation on outcomes in haemodialysis patients

Study	Participants	Design	Intervention	Outcomes
Jamison 2007 [34]. USA	*N* = 2056 (63 % ACKD, 37 % ESKD) (98 % male); Intervention: *n* = 1032 (65 ± 12 years); Placebo: *n* = 1024 (66 ± 12 years)	3-month RCT with 3.2-year follow-up (median)	Daily: 100 mg vitamin B6, 40 mg folate, and 2 mg vitamin B12	↑ folate (short term and long term); ↓ homocysteine (short term and long term); no effect on risk of MI, stroke, amputation or all-cause mortality
Heinz 2010 [33]. Germany	*N* = 650 (58 % male); Intervention: *n* = 327 (61 ± 13 years); Placebo: *n* = 323 (61 ± 13 years)	RCT, median length 2.1 year, range 0.2–5.2 years	3×/week: 20 mg vitamin B6, 5 mg folate, 20 μg vitamin B12	Unstable angina: HR 0.32 (95 % CI 0.12–0.89); no effect on risk of fatal and non-fatal cardiovascular events, and all-cause mortality

*Abbreviations*: *ACKD* Advanced chronic kidney disease, *CI* Confidence interval, *ESKD* End-stage kidney disease, *HR* Hazard ratio, *MI* Myocardial infarction, *RCT* Randomised controlled trial

## Discussion

Cardiovascular complications are the most common cause of mortality in people with ESKD. Given that the Mediterranean diet appears to have cardioprotective properties in the general population, it is feasible that this dietary approach may also be beneficial in this patient population. However, people living with ESKD have altered metabolic processes and fluid and electrolyte balance, causing their uraemic symptoms. Therefore, the beneficial effects of interventions in the general population may not always be true in this group. In the 4D and AURORA studies, for example, common LDL cholesterol-lowering drugs failed to reduce the risks of cardiovascular events in haemodialysis patients, despite being successful in the general population [36, 37].

The extant research suggests that those with higher fish intakes tend to have more favourable albumin and HDL cholesterol levels, as well as a lower risk of mortality compared to those with lower fish intake. Increased levels of omega-3 fatty acids in the blood also appear to lower risk of mortality. However, randomised controlled trials of supplementation with omega-3 fatty acids have assessed different markers of inflammation and nutritional status thereby limiting conclusions. The majority of studies included in this review had small sample sizes and only a few studies were adequately powered randomised controlled trials. Intervention studies involving robust methodology are required to properly examine the physiological mechanisms of fish oil, and its associations with mortality, morbidity and other physiological markers.

Only two studies have examined the effects of vitamin B supplementation on cardiovascular events and mortality in haemodialysis patients, with both showing no effect. Likewise studies examining the role of vitamin E supplementation in haemodialysis patients have had inconsistent results and small sample sizes. This is likely due to methodological differences in dose, route of administration, and treatment duration. Randomised controlled trials of vitamin supplementation should involve combinations of multiple relevant vitamins, rather than just one vitamin in isolation, to examine the synergistic complexity of the antioxidant system. Investigating the cardioprotective role of supplementation with whole foods including fish, fruit and vegetables in these patients, a group with a high medication load, may also provide an alternative avenue of research aimed at improving cardiovascular health in people with ESKD.

## Abbreviations

ACKD: Advanced chronic kidney disease
CI: Confidence interval
CRP: C-reactive protein
CVD: Cardiovascular disease
DHA: Docosahexaenoic acid
DNA: Deoxyribonucleic acid
eGFR: estimated glomerular filtration rate
EPA: Eicosapentaenoic acid
ESKD: End-stage kidney disease
Hb: Haemoglobin content
HD: Haemodialysis
HDL: High density lipoprotein
HR: Hazard ratio
IL-6: Interleukin-6
IU: International units
LDL: Low-density lipoprotein
MI: Myocardial infarction
OR: Odds ratio
PD: Peritoneal dialysis
PUFA: Polyunsaturated fatty acid
RBC: Red blood cell
RCT: Randomised controlled trial
RR: Relative risk
SOD: Superoxide dismutase
TIBC: Total iron binding capacity
TNFα: Tumour necrosis factor-α
WCC: White cell count
